# Co-expression analysis revealed PTCH1-3'UTR promoted cell migration and invasion by activating miR-101-3p/SLC39A6 axis in non-small cell lung cancer: implicating the novel function of PTCH1

**DOI:** 10.18632/oncotarget.23219

**Published:** 2017-12-13

**Authors:** Xuechao Wan, Zhe Kong, Kaili Chu, Chuanyou Yi, Jian Hu, Rui Qin, Chen Zhao, Fangqiu Fu, Hai Wu, Yao Li, Yan Huang

**Affiliations:** ^1^ State Key Laboratory of Genetic Engineering, Shanghai Engineering Research Center of Industrial Microorganisms, School of Life Sciences, Fudan University, Shanghai 200433, PR China

**Keywords:** PTCH1-3'UTR, metastasis, miR-101-3p, WGCNA, non-small cell lung cancer

## Abstract

Metastasis is the most common cause of mortality for non-small cell lung cancer (NSCLC). PTCH1, a receptor of Hedgehog (Hh) pathway, is reported to suppress cell proliferation. Interestingly, our previous study showed PTCH1 silencing promoted cell proliferation but inhibited cell migration and invasion of NSCLC cells. However, the precise mechanisms of PTCH1 regulating NSCLC metastasis remain unclear. PTCH1 has multiple splicing variants, which all share the same 3’UTR sequence, meanwhile, emerging studies have shown competing endogenous RNAs (ceRNAs) play important roles in regulating cancer progression. Therefore, we hypothesized the functions of PTCH1-3’UTR in NSCLC in present study to reveal its role as a ceRNA. Here, we find overexpression of PTCH1-3’UTR promotes cell migration, invasion and adhesion, but does not affect cell proliferation in NSCLC cells. By combining weighted correlation network analysis (WGCNA) analysis and experimental validation, we reported PTCH1-3’UTR acted as a sponge to absorb miR-101-3p and promoted SLC39A6 expression. Moreover, we observed low expression of miR-101-3p and PTCH1 and high SLC39A6 levels were positively correlated with NSCLC progression. Therefore, our results help to understand the function of PTCH1 in NSCLC tumorigenesis and provide novel insights for the prevention of NSCLC metastasis.

## INTRODUCTION

Lung cancer is the leading cause of cancer-associated mortalities worldwide. Non-small cell lung cancer (NSCLC) constitutes 80% of lung cancer cases. Metastasis is the most common cause of mortality for non-small cell lung cancer (NSCLC). Although the precise mechanisms underlying metastasis remain unclear, studies have provided some information that epithelial-mesenchymal transition (EMT) is involved in metastasis. Recent studies also show that some proteins such as Snail [1] and TWIST1 [2] could regulate EMT. However, there is still an urgent need to identify novel key regulators of regulating NSCLC metastasis.

The Hedgehog (Hh) pathway plays a critical role in embryonic lung growth and morphogenesis [3, 4]. PTCH1, a receptor of Hh pathway, suppresses the pathway via inhibiting SMO, which has been studied in different cell lines and tumors. In previous reports, the functions of PTCH1 were mainly involved in inhibiting cell cycle. Overexpression of PTCH1 could inhibit cell proliferation via suppressing the activation of M-phase promoting factor [5]. Moreover, loss of PTCH1 could promote cell cycle progression via inducing nuclear translocation of CCND1 and CCNB1 [6]. In our previous report, we found that PTCH1 silencing promoted cell proliferation of NSCLC cells, but we also found knockdown of PTCH1 significantly inhibited cell migration and invasion [7]. Interestingly, Sheng et al. reported PTCH1 was overexpressed in metastatic prostate cancer compared with normal tissue [8]. These results indicate that PTCH1 might also act as a promoter of metastasis. However, little was known about the role of PTCH1 in tumor migration and invasion.

MicroRNAs (miRNAs) are a class of well-conserved small noncoding RNAs (20-22 nucleotides long) [9, 10], which regulate gene expression mainly through binding to the 3'-untranslated region (3'UTR) of target transcripts [9, 11]. Recently, emerging evidences suggest that 3'UTR of genes could function as competing endogenous RNAs (ceRNAs to regulate other RNA transcripts by competing for shared miRNAs. For example, TP53INP1 3’UTR could inhibit the EMT via acting as a ceRNA for E-cadherin [12]. Zheng et al. also reported CXCR4 3’UTR functioned as a ceRNA in promoting metastasis and proliferation of MCF-7 cells by regulating miR-146a activity [13]. The finding provided a new insight to molecular function of mRNA besides the protein-coding function. Of note, PTCH1 has multiple splicing isoforms, but they all share a same 3'-UTR sequence, which indicates the importance of PTCH1 3’UTR.

In the present study, we focused on the role of PTCH1-3’UTR in NSCLC. We found that overexpression of PTCH1 3’UTR promoted cell migration, invasion and adhesion, but did not affect cell proliferation in NSCLC cells. SLC39A6, a regulator of metastasis, was identified as downstream of PTCH1-3’UTR. We identified the microRNA responsive elements (MREs) for miR-101-3p in both PTCH1- and SLC39A6- 3’UTR. Accordingly, we reported a novel mechanism driving metastasis mediated by PTCH1 whose 3’UTR acted as a sponge to absorb miR-101-3p and promoted SLC39A6 expression.

## RESULTS

### Overexpression of PTCH1 3’UTR promotes cell migration, invasion and adhesion, but has no effect on cell proliferation

In our previous study, we found PTCH1 silencing promoted cell proliferation, but inhibited cell migration and invasion in NSCLC cell lines. Considering that multiple splicing isoforms of PTCH1 shared the same 3’UTR, thus, we hypothesized that PTCH1 might promote NSCLC metastasis via its 3’UTR. To test this, we transfected pcDNA3.1-PTCH1-3’UTR into NSCLC cells and performed a series of cell function assays.

**Figure 1:**
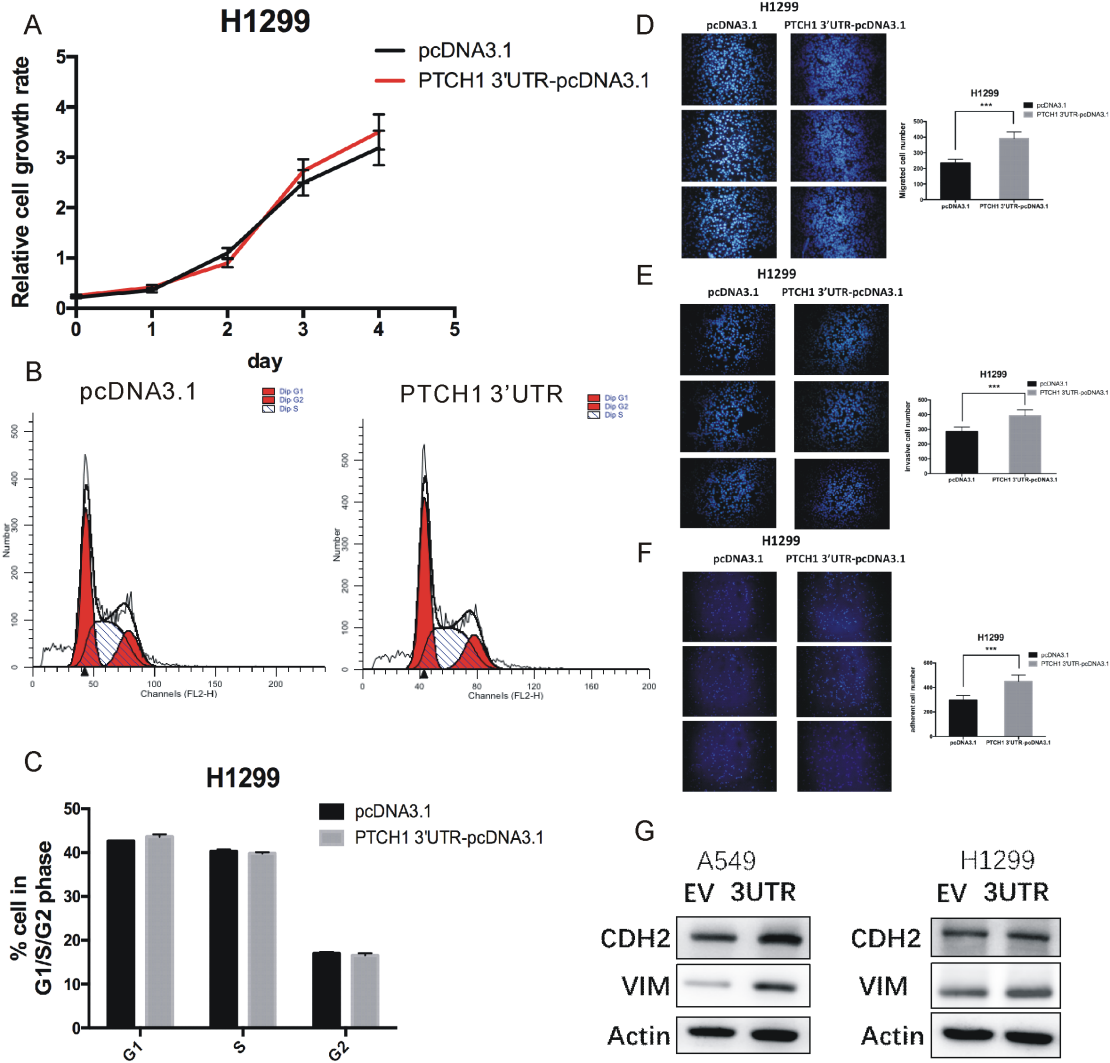
Overexpression of PTCH1 3’UTR promotes cell migration, invasion and adhesion in H1299 **(A)** Cell proliferation analysis was performed with CCK-8 assay in H1299 cells. Cells transfected with PTCH1 3’UTR or pcDNA 3.1 vector were seeded into 96-well plate at 5000 cells/well and examined at time points of 0h, 24h, 48h, 72 h and 96h. Overexpression of PTCH1 3’UTR had no significant difference with that transfected with pcDNA3.1. **(B-C)** Cell cycle assay was performed in H1299 cells. Cells were transfected with PTCH1 3’UTR or pcDNA 3.1 vector for 48 h, stained with PI and evaluated with a FACScalibur flow cytometer. Overexpression of PTCH1 3’UTR had almost the same population of cells in the G1 (resting), S (synthesis) and G2 (mitotic) phases with negative control. **(D-F)** Migrations, invasion and adhesion assay were performed in H1299 cells. Cells were transfected with PTCH1 3’UTR or control and seeded into the upper chamber of a transwell to count by transwell assay. Overexpression of PTCH1 3’UTR promotes cell migration, invasion and adhesion compared with negative control. Data are presented as the mean ± SD (n = 3). Significance was defined as p<0.05 (^*^, p < 0.05; ^**^, p < 0.01; ^***^, p < 0.001). **(G)** Effect of PTCH1 3’UTR on the protein expression levels of CDH2 and VIM in A549 and H1299 cells using western blot analysis. β-actin was used as internal control.

We first conducted CCK-8 assay to assess the cell growth rates of NSCLC cells. Our results demonstrated that the proliferation rate of H1299 and A549 cells transfected with pcDNA3.1-PTCH1 3’UTR had no significant difference compared with the cells transfected with pcDNA3.1 (Figure 1A and Supplementary Figure 1A). Meanwhile, cell-cycle analysis showed that cells overexpressed PTCH1-3’UTR had almost the same population of cells in the G1 (resting), S (synthesis) and G2 (mitotic) phases (Figure 1B-1C and Supplementary Figure 1B-1C) compared to that of the control cells, which were consistent with the results of cell proliferation viability assay.

Metastasis is a process that involves multiple steps, including cancer cell adhesion, invasion and migration [14]. To study the roles of PTCH1-3’UTR in these processes, we overexpressed PTCH1-3’UTR in H1299 and A549 cells. 48 hour post-transfection, adhesion, migration, and invasion assays were performed. We observed overexpression of PTCH1-3’UTR promoted cell migration by 66% in H1299 compared with the cells transfected with pcDNA3.1 (Figure 1D). Cells overexpressed PTCH1-3’UTR also showed an increase in cell invasion and cell adhesion by about 37% and 50%, respectively (Figure 1E-1F). Furthermore, we performed these experiments in A549 cells and found the similar results (Supplementary Figure 1D-1F). These results indicated PTCH1-3’UTR significantly increased adhesion, invasion, and migration of NSCLC cells.

Vimentin is the major cytoskeletal component of mesenchymal cells, which is often used as a marker of mesenchymal-derived cells or cells undergoing an EMT during cancer metastatic progression [15]. N-cadherin (CDH2) functions to mediate cell–cell adhesion, it also plays a role in cancer metastasis. To further confirm that PTCH1-3’UTR upregulated cell metastasis, we examined the expression of these two proteins in A549 and H1299 cells. Western blotting revealed that PTCH1-3’UTR transient transfected H1299 and A549 cells both showed enhanced vimentin levels and CDH2 levels compared with control cells (Figure 1G). Taken together, these results suggested that PTCH1 3’UTR did not affect cell proliferation, but promoted cell metastasis in NSCLC cells.

### Weighted gene network co-expression analysis of genes shared same MREs with PTCH1

**Figure 2:**
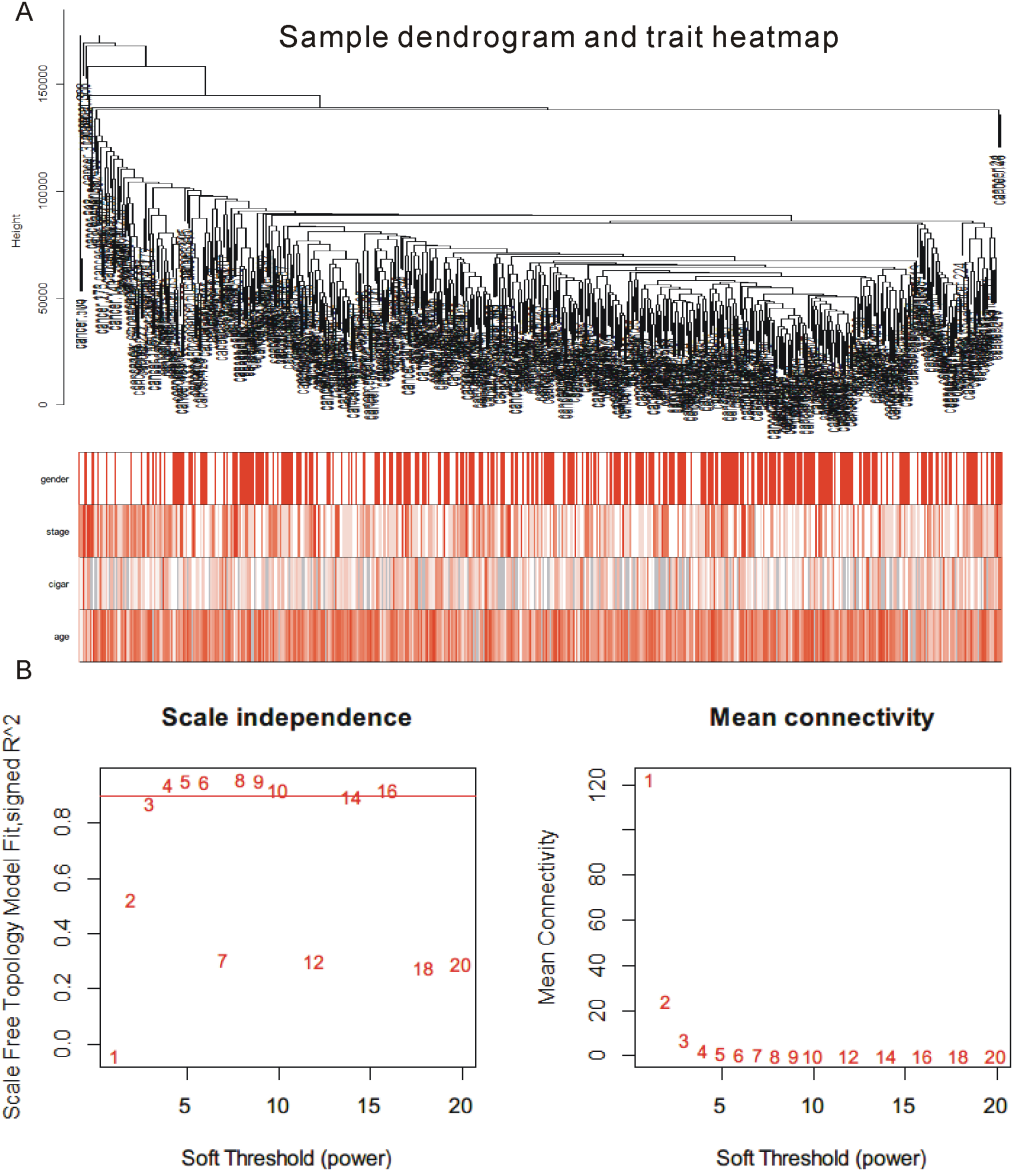
Result of WGCNA analysis **(A)** Cluster result and trait heatmap of data samples. **(B)** Determination of parameter β of the adjacency function in the weighted gene correlation network analysis (WGCNA) algorithm.

Weighted correlation network analysis (WGCNA) has been successfully applied to cancer-related studies. In this study, we applied WGCNA [16, 17] to explore the potential mechanisms of PTCH1-3’UTR-mediated-metastasis using TCGA lung cancer data, which included 517 NSCLC samples. A total of 1095 differentially expressed genes between normal tissues and tumor samples in TCGA data sharing the same MREs as PTCH1 were selected for further study. Figure 2A showed the cluster of the 517 samples, and no abnormal sample was found. The stratification according to gender, stage, smoking status, as well as age were shown to indicate the clinic characterize of these samples. The co-expression network was constructed with WGCNA package in the R software. The results of the parameter analysis were shown in Figure 2B. After determining the optimal parameter (β=4), the WGCNA algorithm was used to convert the correlation coefficient of a gene pair into the adjacent coefficient.

**Figure 3:**
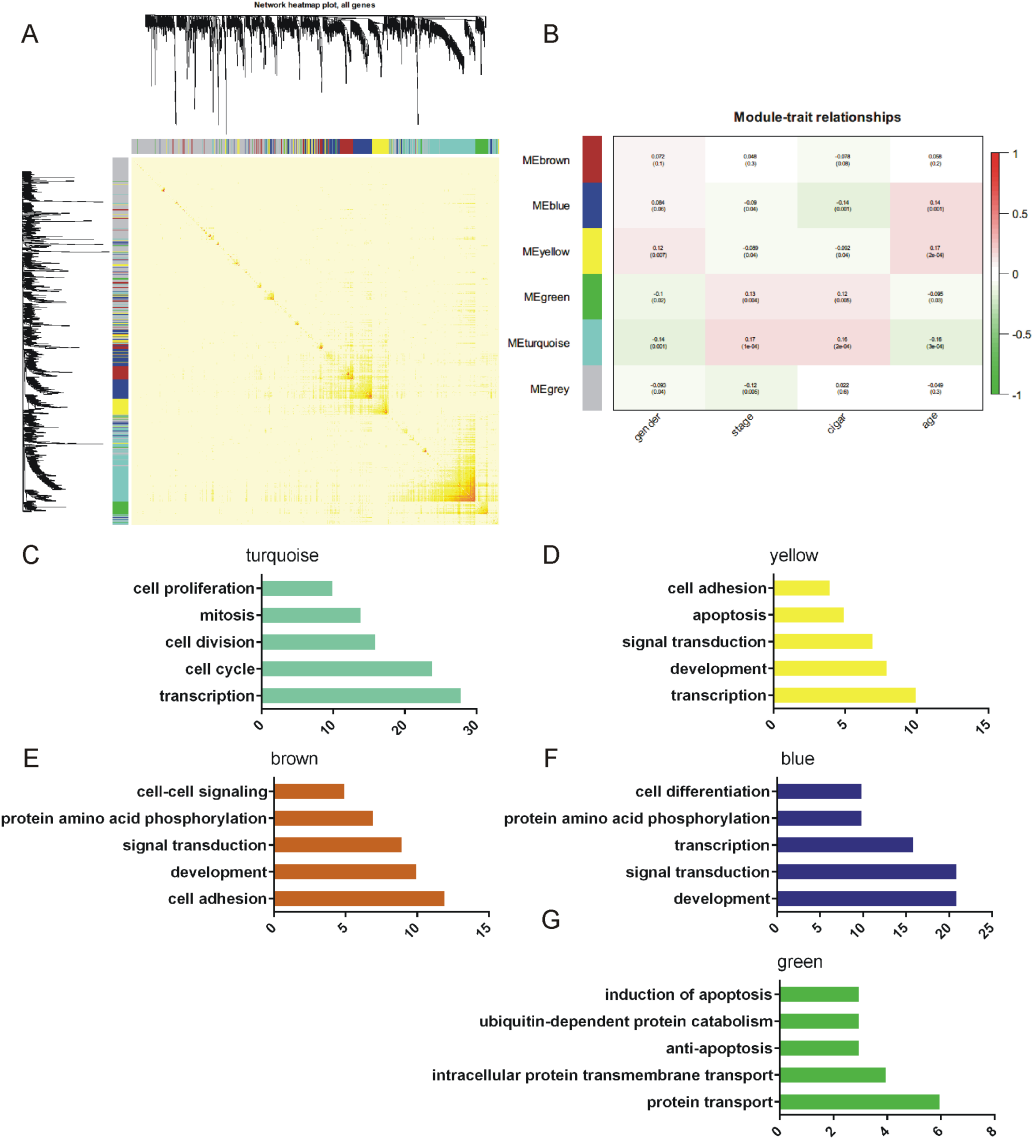
Construction of the gene co-expression network GO analysis of these modules. **(A)** Construction of the gene co-expression network. Each color represents a certain gene module. **(B)** The association between each module and the clinical character. The resulting module-clinic correlation was visualized as a heatmap in (B). **(C-G)** Top 5 of biological processes in GO analysis of turquoise (C), yellow (D), brown (E), blue (F), and green (G) module.

Our analysis classified the 1095 differentially expressed genes that shared the same MREs as PTCH1 into 5 modules, and each was assigned to a unique color. Turquoise, blue, brown, yellow, and green represented 241, 154, 96, 91, and 64 genes, respectively. Among these genes, 449 genes were not assigned to any of the 5 modules, which designated as grey (Figure 3A). A complete list of the network metrics and the module membership for each gene were shown in Supplementary Table 3.

The association between each module and the clinical character were then analyzed. The resulting module-clinic correlation was visualized as a heatmap (Figure 3B). We found that genes clustered in turquoise showed the most significant correlation with the disease state. Based on the correlation co-efficient, we observed genes in blue and yellow modules were positive-related to age and negative-related to stage, while genes in green and turquoise modules were positive-related to stage, and negative-related to age (Figure 3B).

### GO analysis of the differential expressed genes sharing the same MREs as PTCH1

In order to explore the potential functions of the differential expressed genes sharing the same MREs as PTCH1, we performed GO analysis of the genes clustered in modules (Figure 3C-3G). According to the GO analysis, genes in turquoise modules were enriched in transcription, cell cycle, cell division, mitosis, cell proliferation (Figure 3C). Genes in yellow modules were enriched in transcription, development, signal transduction, apoptosis, cell adhesion (Figure 3D). Genes in brown modules were enriched in cell adhesion, development, signal transduction, protein amino acid phosphorylation, cell-cell signaling (Figure 3E). Genes in blue modules were enriched in development, signal transduction, transcription, protein amino acid phosphorylation, cell differentiation (Figure 3F). Genes in green modules were enriched in protein transport, intracellular protein transmembrane transport, anti-apoptosis, ubiquitin-dependent protein catabolism and induction of apoptosis (Figure 3G). These results suggested genes in brown modules might be the most significantly related to the function of PTCH1-3’UTR in regulating metastasis, since development and cell-to-cell adhesion are associated with metastasis.

### PTCH1 3’UTR up-regulated SLC39A6 expression in NSCLC cells

**Figure 4:**
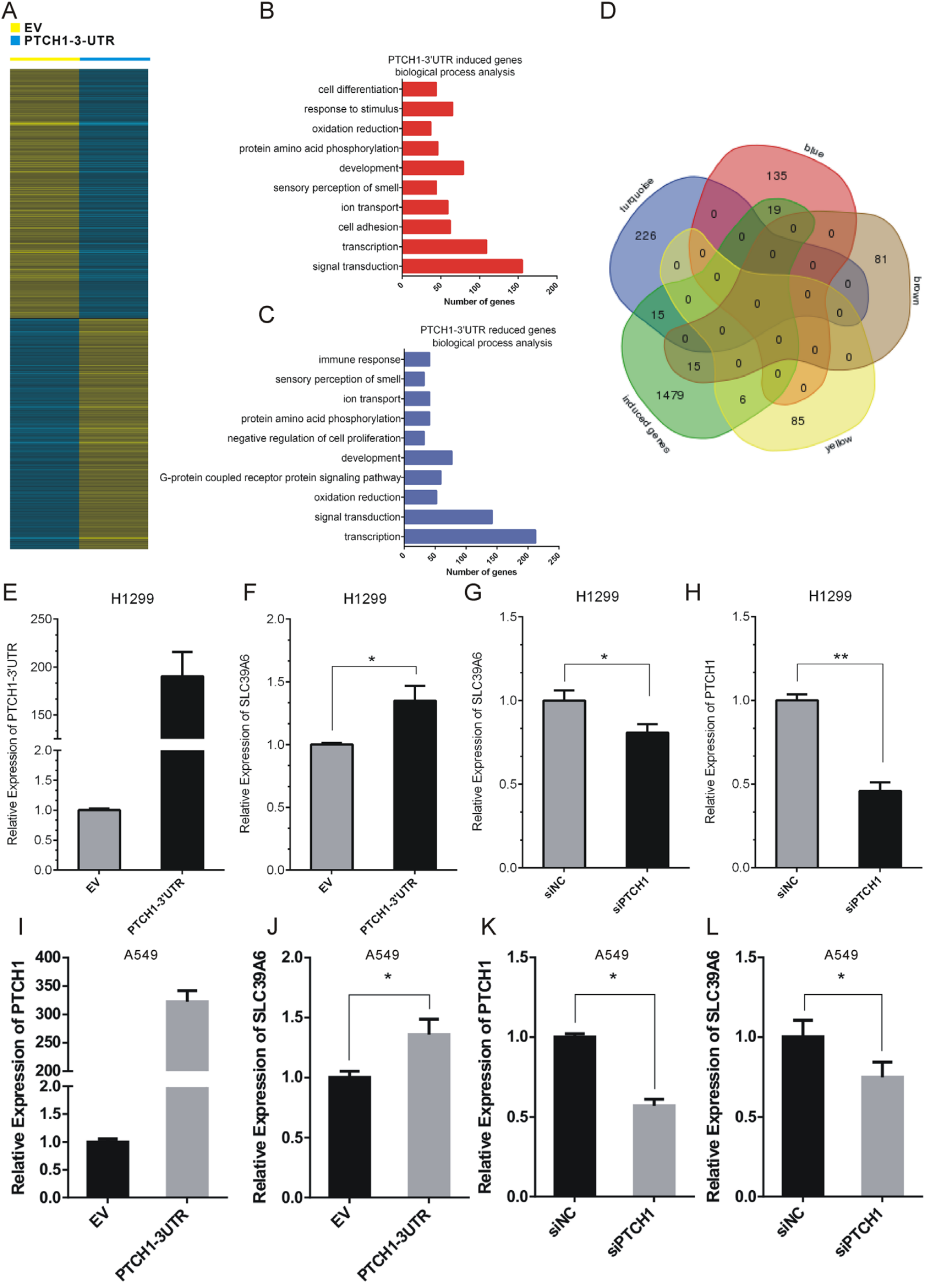
PTCH1 3’UTR up-regulated SLC39A6 expression in NSCLC cells **(A)** Identification of PTCH1 3’UTR regulated genes in H1299 cells. **(B-C)** GO analysis of up-regulated transcripts (B) and down-regulated (C) transcripts of PTCH1 3’UTR. Number of genes is indicated at x-axis. Up-regulated genes were enriched in the biological processes of signal transduction, cell adhesion and cell differentiation. Down-regulated genes were enriched in the biological processes of transcription, signal transduction and development. **(D)** Venn diagrams for combing the PTCH1 3’UTR inducing genes with previous module genes. 15 percent of genes in brown modules (15/96) were up-regulated after overexpressing PTCH1 3’UTR. **(E-H)** RT-PCR analysis of SLC39A6 and PTCH1 expression level after overexpression PTCH1-3’UTR or silencing PTCH1 in H1299 cells. **(I-L)** RT-PCR analysis of SLC39A6 and PTCH1 expression level after overexpression PTCH1-3’UTR or silencing PTCH1 in A549 cells. Data are presented as the mean ± SD (n = 3). Significance was defined as p<0.05 (^*^, p < 0.05; ^**^, p < 0.01; ^***^, p < 0.001).

To obtain insight into the potential biological process and molecular pathways affected by PTCH1-3’UTR, we used Agilent Human lncRNA array to screen the PTCH1 3’UTR regulated mRNA. H1299 cells were transiently transfected with pcDNA3.1-PTCH1-3’UTR or pcDNA3.1 empty vector for 48 hour, and the total RNA was extracted and reverse transcribed. Supervised analysis of the microarray data showed 1991 up-regulated transcripts and 1776 down-regulated transcripts, with an average expression level over 1.5-fold change compared with the control group (Figure 4A). These genes were mapped using the KEGG pathway database and BioCarta pathway database.

Our analysis revealed that up-regulated genes strongly overlapped with genes that regulated signal transduction, cell adhesion, and cell differentiation, while down-regulated genes strongly overlapped with genes that regulated G-protein coupled receptor protein signaling pathway, immune response and oxidation reduction (Figure 4B-4C). By combing the PTCH1-3’UTR inducing genes with the module genes, we found that 15 percent of genes in brown modules (15/96) were up-regulated after overexpressing PTCH1-3’UTR, from which SLC39A6 was selected for further validation.

To evaluate that PTCH1-3’UTR affected the expression of SLC39A6, we examined the mRNA lever of SCL39A6 after overexpressing PTCH1-3’UTR or knockdown PTCH1 in H1299 and A549 cells. Transfection efficiency were shown in Figure 4E, 4G, 4I and 4K. Our results showed that PTCH1-3’UTR could increase SLC39A6 expression by about 40% in H1299 and A549 cells (Figure 4F and 4J). However, knockdown of PTCH1 inhibited SLC39A6 expression by about 30% in H1299 and A549 cells (Figure 4H and 4L).

### PTCH1 and SLC39A6 were direct targets of miR-101-3p

**Figure 5:**
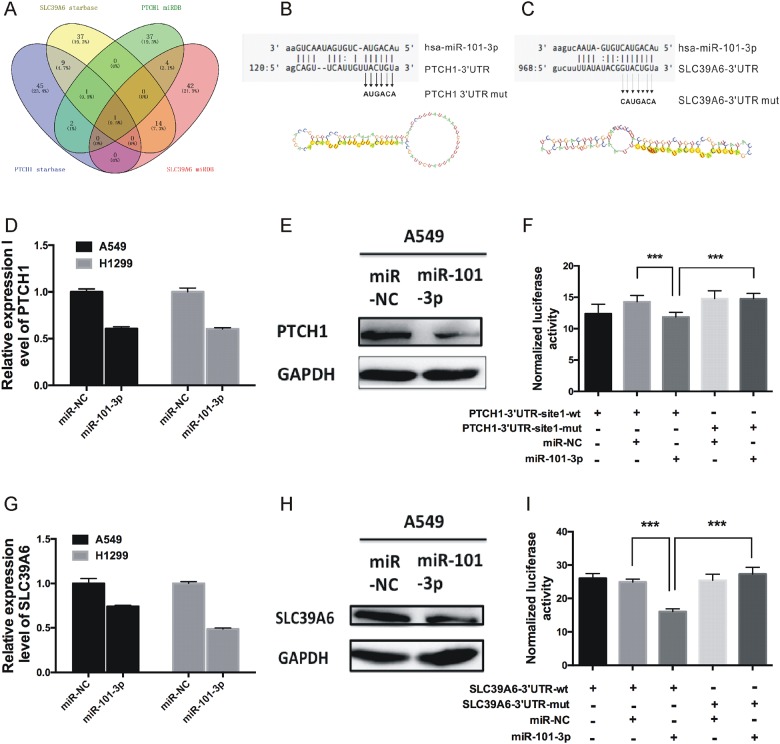
PTCH1 and SLC39A6 were direct targets of miR-101-3p **(A)** Venn diagrams for miRNAs binding to both PTCH1 and SLC39A6 using two open-access, including miRDB and CLIP-seq database. **(B-C)** Sequence alignment of human miR-101-3p and 3’-UTR of PTCH1 and SLC39A6 using TargetScan and microRNA.org. The seed sequence of miR-101-3p (top) matches 3’-UTR of PTCH1(B, middle) and SLC39A6(C, middle). Bottom is the mutations of the 3’-UTR of PTCH1 and SLC39A6 used in luciferase reporter construct. **(D-E** and **G-H)** RT-PCR and western blotting analysis of PTCH1 and SLC39A6 mRNA and protein expression levels after overexpression miR-101-3p in A549 and H1299. GAPDH was used as internal control. **(F, I)** MiR-101-3p overexpression decrease the luciferase activity of PTCH1 and SLC39A6, but did not affect that of PTCH1-mut and SLC39A6-mut. Data are presented as the mean ± SD (n = 3). Significance was defined as p<0.05 (^*^, p < 0.05; ^**^, p < 0.01; ^***^, p < 0.001).

We performed a computational screen for miRNAs with complementary sites to PTCH1 or SLC39A6 in their binding sites using open-access software including miRDB (http://mirdb.org/cgi-bin/search.cgi). Also, we further used online CLIP-seq database to identify miRNAs that interact with PTCH1 and SLC39A6 (http://starbase.sysu.edu.cn/) (Figure 5A). From both analyses, miR-101-3p was shown to potentially bind to PTCH1, as well as SLC39A6 (Figure 5B-5C).

To test PTCH1 and SLC39A6 were direct targets of miR-101-3p, we first transiently transfected miR-101-3p in A549 and H1299 cells, and the expression of PTCH1 and SLC39A6 mRNA and protein were examined by real-time PCR and western blotting. Consistent with our prediction, overexpression of miR-101-3p decreased PTCH1 and SLC39A6 mRNA levels in A549 and H1299 cells (Figure 5D and 5G). Western blot assay showed PTCH1 and SLC39A6 protein levels were also decreased significantly in A549 (Figure 5E and 5H) and H1299 (Supplementary Figure 2A-2B) cells transfected with miR-101-3p compared with cells transfected with miR-NC.

The binding of miR-101-3p to PTCH1- and SLC39A6-3’UTR were detected by luciferase assays. For this purpose, we created a pair of luciferase reporter constructs containing either the wild type (wt) or the mutant (mut) in the binding site of miR-101-3p to PTCH1 and SLC39A6-3’UTR. The luciferase reporter assay revealed that luciferase activity was significantly repressed in the constructs of the PTCH1-3’UTR (Figure 5F) and SLC39A6-3’UTR (Figure 5I) when co-transfected with the corresponding miRNAs compared with NC, whereas the mutated 3’UTR did not show a significant response to miR-101-3p.

### MiR-101-3p suppressed proliferation, migration and invasion of NSCLC cells

**Figure 6:**
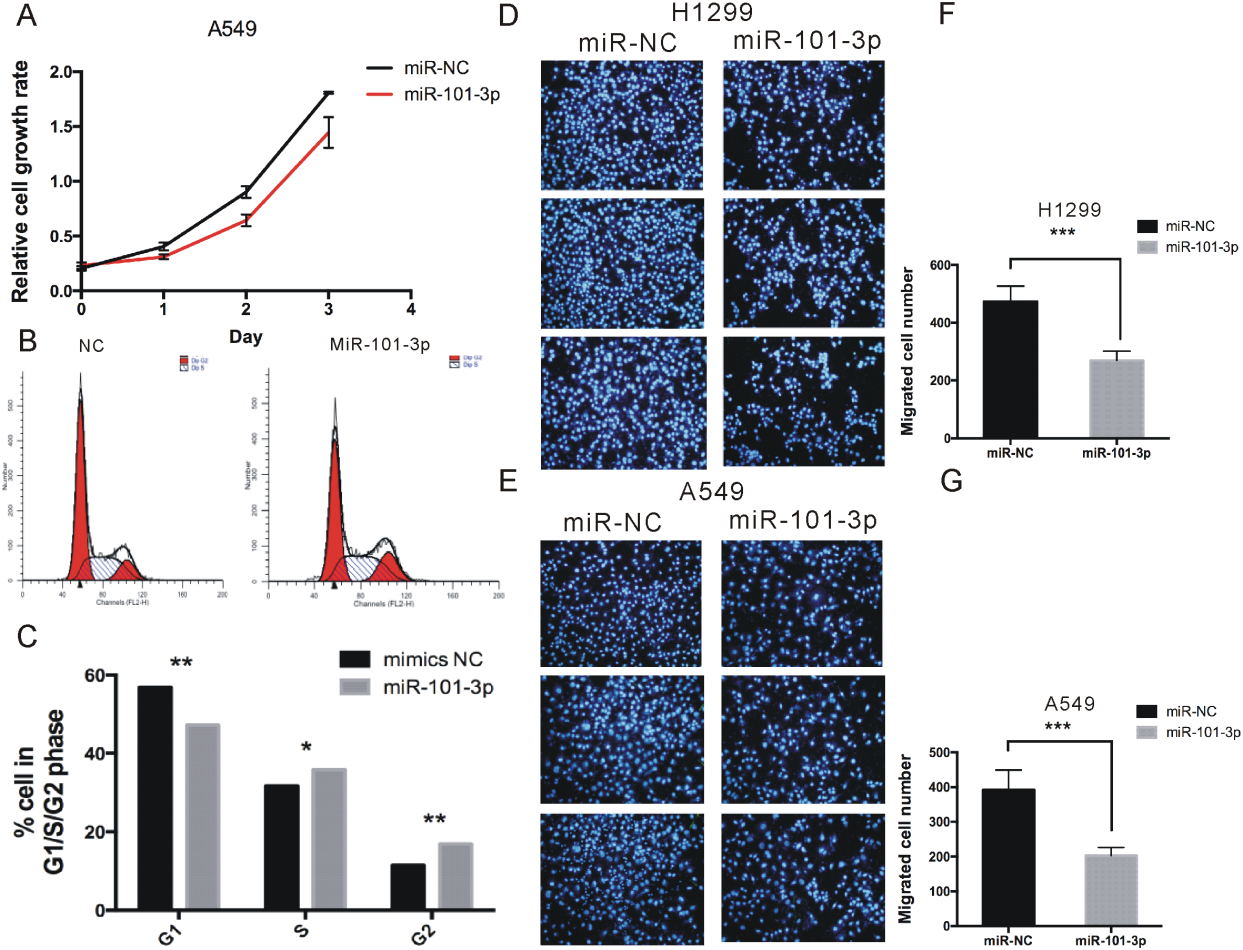
miR-101-3p suppresses proliferation, migration and invasion of NSCLC cells **(A)** Cell proliferation analysis was performed with CCK-8 assay in A549 cells. Cells transfected with miR-101-3p or miR-NC were seeded into 96-well plate at 5000 cells/well and examined at time points of 0h, 24h, 48h and 72h. Overexpression of miR-101-3p significantly suppressed proliferation compared with that transfected with miR-NC. **(B-C)** Cell cycle assay was performed. Cells were transfected with miR-101-3p or miR-NC for 48 h, stained with PI and evaluated with a FACScalibur flow cytometer. Overexpression of miR-101-3p significantly induced the population of cells in the S/G1 compared with negative control. **(D-G)** Migrations assay were performed in H1299 and A549 cells respectively. Cells were transfected with miR-101-3p or miR-NC and seeded into the upper chamber of a transwell to count by transwell assay. Overexpression of miR-101-3p promotes cell migration compared with negative control. Data are presented as the mean ± SD (n = 3). Significance was defined as p<0.05 (^*^, p < 0.05; ^**^, p < 0.01; ^***^, p < 0.001).

To evaluate the biological functions of miR-101-3p in NSCLC, we conducted gain of function studies by transient transfection with miR-101-3p mimics in A549 and H1299 cells. We assessed cell growth rate by CCK-8 assay to study the effect of miR-101-3p on cell proliferation in A549 and H1299. Compared with miR-NC, miR-101-3p mimics significantly inhibited cell proliferation (Figure 6A and Supplementary Figure 2C). To further study the mechanism by which miR-101-3p affected proliferation, cell cycle progression was analyzed using flow cytometry. The results showed that overexpression of miR-101-3p exhibited a longer S/G2 phase compared with corresponding control group (Figure 6B-6C).

To study the role of miR-101-3p in migration, we overexpressed miR-101-3p in A549 and H1299 cell lines. 48 h post-transfection, migration assays were performed. As shown in Figure 6D-6G, miR-101-3p significantly inhibited migration of A549 and H1299 cell by 48% and 43% compared with corresponding control group, respectively.

### SLC39A6 mediated the effect of PTCH1-3’UTR on NSCLC cell migration and invasion

**Figure 7:**
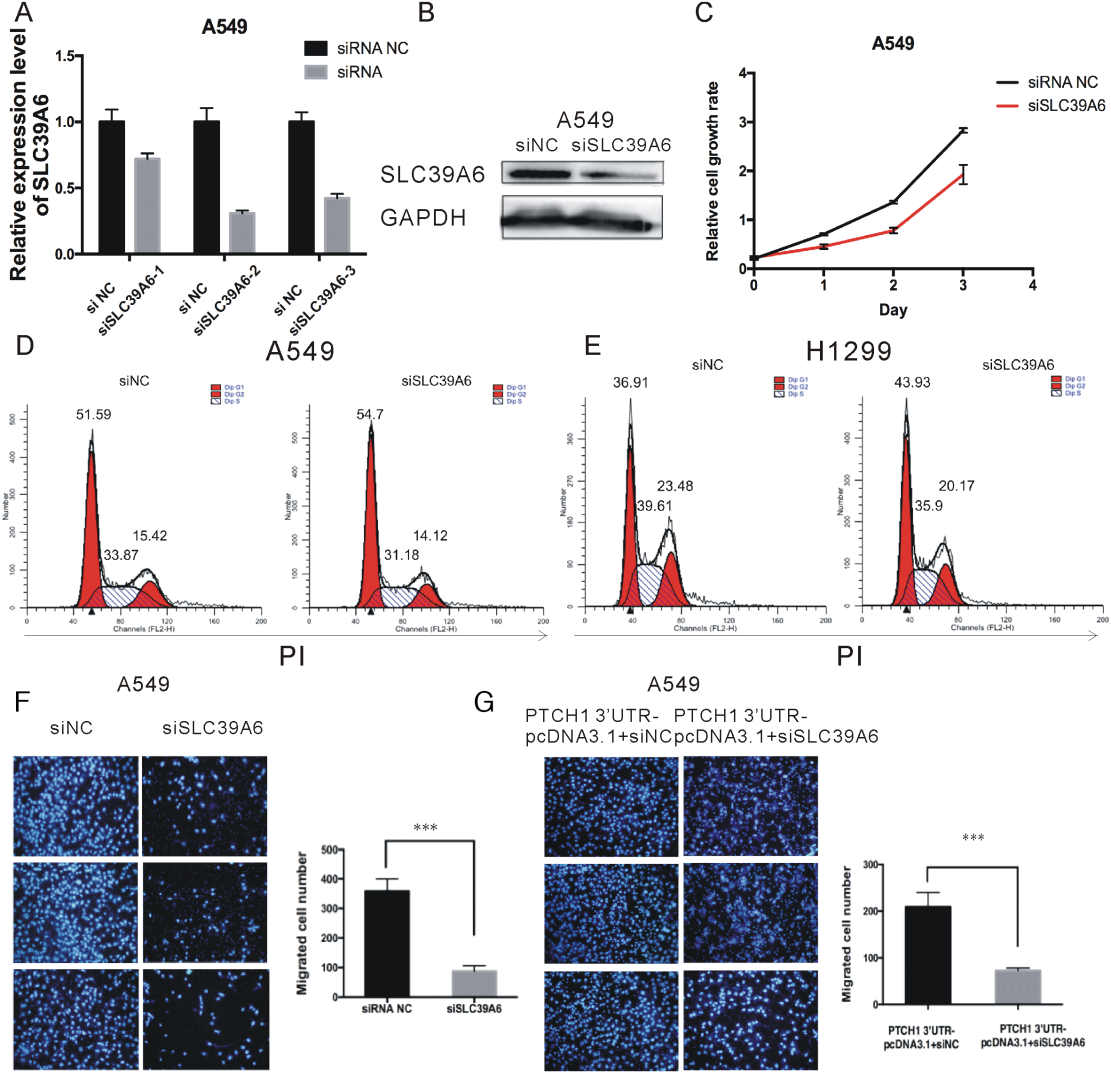
PTCH1 3’UTR mediated the effect of SLC39A6 on NSCLC migration **(A-B)** RT-PCR and western blot analysis of SLC39A6 expression level after knockdown of SLC39A6 by silencing RNA. **(C)** Cell proliferation analysis was performed with CCK-8 assay in A549 cells. Cells transfected with siSLC39A6 or negative control were seeded into 96-well plate at 5000 cells/well and examined at time points of 0h, 24h, 48h and 72h. Knockdown of SLC39A6 significantly suppressed proliferation compared with that transfected with negative control. **(D-E)** Cell cycle assay was performed in H1299 and A549 respectively. Cells were transfected with siSLC39A6 or siNC for 48 h, stained with PI and evaluated with a FACScalibur flow cytometer. Knockdown of SLC39A6 significantly induced an increase in G1 phase and a decrease in S phase compared with negative control. **(F)** Migrations of A549 cells after siSLC39A6 transfection were counted by transwell assay, knockdown of SLC39A6 significantly decreased cell migration compared with negative control. **(G)** Migrations of A549 cells after PTCH1-3’UTR-siSLC39A6 co-transfection were counted by transwell assay, in the presence of PTCH1-3’UTR, the effect of siSLC39A6 on migration could be partially rescued. Data are presented as the mean ± SD (n = 3). Significance was defined as p<0.05 (^*^, p < 0.05; ^**^, p < 0.01; ^***^, p < 0.001).

SLC39A6, also named LIV-1, is a zinc transporter that regulates the invasion and metastasis of pancreas, esophageal and prostate cancers [18–20]. However, the function of SLC39A6 in NSCLC metastasis remains unknown. Therefore, we investigated the role of SLC39A6 in cell migration and invasion using siRNA against SLC39A6 in NSCLC cells (Figure 7A-7B and Supplementary Figure 3A). Proliferation assays showed SLC39A6 knockdown inhibited cell proliferation rate by about 30% compared with the controls (Figure 7C and Supplementary Figure 3B). The cell cycle analyses also indicated that cells transfected with siSLC39A6 showed a delay in G1 cells in both H1299 and A549 cells (Figure 7D-7E).

To show whether SLC39A6 was involved in PTCH1-3’UTR-mediated cell migration and invasion, A549 cells were transfected with siSLC39A6 or siRNA NC, and cell migration ability was assessed. Cell migration was significantly decreased by 77% in siSLC39A6-transfected cells compared to that of the control cells (Figure 7F). And then, PTCH1-3’UTR was transfected into the SLC39A6 silenced cells. The result showed that in the presence of PTCH1-3’UTR, the effect of SLC39A6 siRNA on migration could be partially rescued. A549 cells co-transfected with PTCH1-3’UTR and siSLC39A6 attenuated cell migration by 65%, which led to about 12% increase of cell migration rate compared to the cells only transfected with siSLC39A6(Figure 7G). Of note, we got the similar results by using H1299 cells (Supplementary Figure 3C and 3D). Hence, we found that PTCH1-3’UTR had the impact on SLC39A6 regarding to cell migration.

### Low miR-101-3p and PTCH1 and high SLC39A6 levels were positively correlated with NSCLC progression

**Figure 8:**
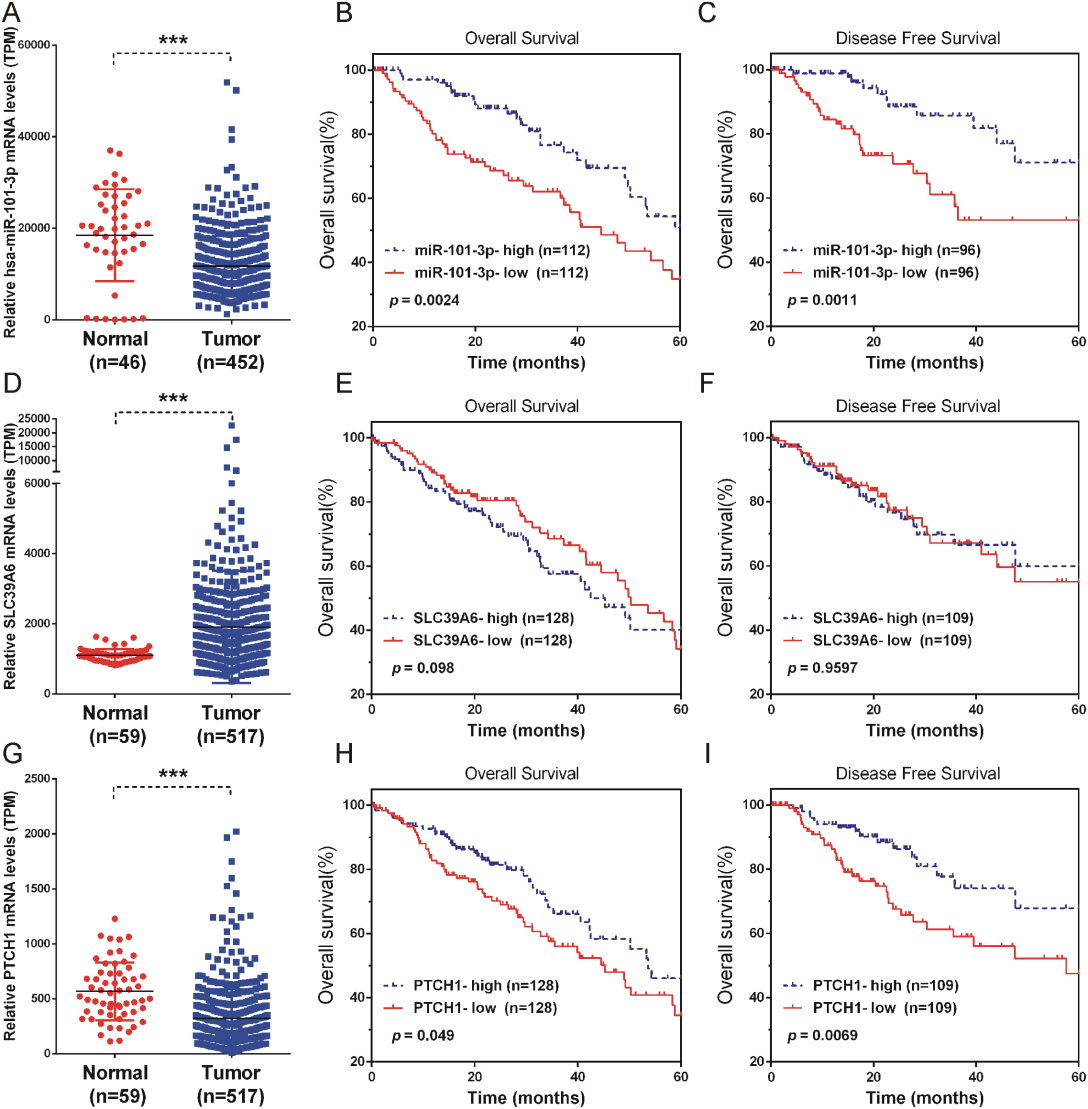
Low miR-101-3p and PTCH1 and high SLC39A6 levels were positively correlated with NSCLC progression **(A, D, G)** The expression of miR-101-3p, SLC39A6 and PTCH1 in NSCLC samples (n=452) compared to normal tissues (n=46). (**B, C, E, F, H** and **I**) Kaplan-Meier curves for survival time and disease-free survival time in patients with non-small cell lung cancer according to expression of miR-101-3p (B-C), SLC39A6(E-F), PTCH1 (H-I). Significance was defined as p<0.05 (^*^, p < 0.05; ^**^, p < 0.01; ^***^, p < 0.001).

To evaluate possible prognostic value of miR-101-3p, PTCH1-3’UTR and SLC39A6, we analyzed RNA-seq data from TCGA. Since there is no data for any 3’UTR, we examined the PTCH1 instead. As shown in Figure 8, miR-101-3p and PTCH1 were significantly down-regulated, while SLC39A6 was up-regulated in NSCLC samples compared to normal tissues.

Moreover, Kaplan-Meier analysis using log-rank test was performed to assess the potency of miR-101-3p, PTCH1 and SLC39A6 expression on survival of NSCLC patients. Significant correlation between low miR-101-3p and shorter overall survival time/disease-free survival time was found (Figure 8E). A high level of SLC39A6 expression was slightly associated with shorter overall survival time (Figure 8H-8I). However, we did not observe significantly correlation between SLC39A6 expression and disease-free survival time in NSCLC patients (Figure 8F). The results indicated that miR-101-3p and SLC39A6 might be useful prognostic markers for lung cancer patients at all disease stages. Although SLC39A6 and PTCH1-3’UTR could be ceRNA regulated by miR-101-3p, there was a significant correlation between low PTCH1 and shorter overall survival time/disease-free survival time as well, which was different from that of SLC39A6. This may due to PTCH1 mRNA played its role by its encoded protein, together with its non-coding PTCH1-3’UTR.

## DISCUSSION

The well-known function of PTCH1 is acting as a receptor of Hh pathway to suppress the pathway and inhibiting cell proliferation by regulating cell cycle. In our previous report, we found that PTCH1 silencing promoted cell proliferation but inhibited cell migration and invasion of NSCLC cells [7]. To elucidate the mechanisms by which PTCH1 affected metastasis of NSCLC, we hypothesized that the 3’UTR of PTCH1 acted as sponge RNA that attracted many miRNAs to impair miRNAs’ inhibition effect on their original targets, thus promoting metastasis. Of note, recent studies have also revealed that 3’UTR of coding genes could act as ceRNAs to regulate oncogenesis and cancer progression via post-transcriptional regulation [21, 22]. In this study, we overexpressed PTCH1-3’UTR in NSCLC cell lines and found that PTCH1-3’UTR overexpression significantly promoted cell migration, cell invasion and cell adhesion. However, we also observed PTCH1-3’UTR did not affect cell proliferation. These results were consistent with our hypothesis.

To further explore the mechanism of PTCH1-3’UTR regulating metastasis, we performed WGCNA using TCGA data. WGCNA has been successfully applied to cancer-related studies. For example, Zhang et al. identified a novel 22-gene signature of carbon metabolism in hepatocellular carcinoma using gene network analysis [23]. In this study, we first took analysis of 1095 genes with the same MREs as PTCH1, and identified 5 modules, of which 2 modules were involved in cell adhesion regulating. EMT is involved in cancer metastasis, it provides a potential mechanism by which epithelial cancer cells detach from the neighboring cells of the primary tumor, invade through the underlying basement membrane and migrate into the surrounding stroma. Cultures of PTCH1-3’UTR overexpressing H1299 and A549 were less adhered to their neighbor cells, whereas the control cells stably contacted with each other. Therefore, we aimed to search for candidate genes that regulating cell adhesion.

Next, we performed microarray to identify downstream targets of PTCH1-3’UTR. Our results showed that PTCH1-3’UTR up-regulated genes were also involved in cell adhesion regulating. Combining these data, we selected SLC39A6 for further study. Our results showed that PTCH1-3’UTR overexpression could promote SLC39A6 expression in NSCLC cells. To identify potential miRNAs mediating the regulation of SLC39A6 by PTCH1-3’UTR, we performed an in silico analysis, and identified miR-101-3p binding to both genes. In NSCLC cells, overexpression of miR-101-3p reduced PTCH1 and SLC39A6 at both mRNA and protein levels. The luciferase reporter assay revealed that miR-101-3p could significantly decrease luciferase activity in the constructs containing the miR-101-3p binding site of PTCH1 and SLC39A6-3’UTR compared with NC, whereas the mutated 3’UTR did not show a significant response to miR-101-3p. To our knowledge, this is the first report to validate PTCH1 and SLC39A6 are direct targets of miR-101-3p.

Recent studies have shown that miR-101-3p acts as a tumor suppressor and regulating various biological processes, such as tumor metabolism, proliferation and metastasis in different types of tumors [24–26]. In this study, we conducted gain of function studies in NSCLC cells to evaluate the biological functions of miR-101-3p. Our results showed up-regulation of miR-101-3p could inhibit cell proliferation, cell cycle progression, and migration in NSCLC cell lines, suggesting miR-101-3p played a tumor-suppressive role in NSCLC. We also found miR-101-3p was significantly down-regulated in NSCLC samples compared to normal tissues through analyzing the TCGA data. Importantly, better overall survival rates and disease-free survival time of NSCLC patients correlated with higher miR-101-3p expression levels. Thus, miR-101-3p could act as a useful indicator for NSCLC outcomes, which is in consistency with other reports [27].

SLC39A6 regulates the invasion and metastasis of pancreas, esophageal and prostate cancers [18–20]. However, little information regarding the role of SLC39A6 in NSCLC is available. Our study demonstrated that SLC39A6 expression promoted NSCLC cell migration. We knockdown SLC39A6 expression and found SLC39A6 silencing reduced the proliferation and migration ability of NSCLC cells. Importantly, we also found that in the presence of PTCH1-3’UTR, the effect of SLC39A6 siRNA on migration could be partially rescued, suggesting that PTCH1 3’UTR might promote NSCLC metastasis via up-regulating SLC39A6 expression. Moreover, we found SLC39A6 was up-regulated in NSCLC samples in TCGA database.

As described above, we found that PTCH1 inhibited NSCLC cell proliferation and also promoted NSCLC cell metastasis via 3’UTR. TCGA analysis showed PTCH1 was down-regulated in NSCLC samples compared to normal tissues. Better overall survival rates and disease-free survival time of NSCLC patients correlated with higher PTCH1 expression levels, whereas we also found that PTCH1 was significantly up-regulated in metastasis NSCLC samples (data not shown). These results showed two opposite functional roles of PTCH1 both play important roles in regulating NSCLC progression. Thus, understanding the different roles of coding protein and 3’UTR of PTCH1 will lead to novel insight into gene functions and have implications in human disease.

**Figure 9:**
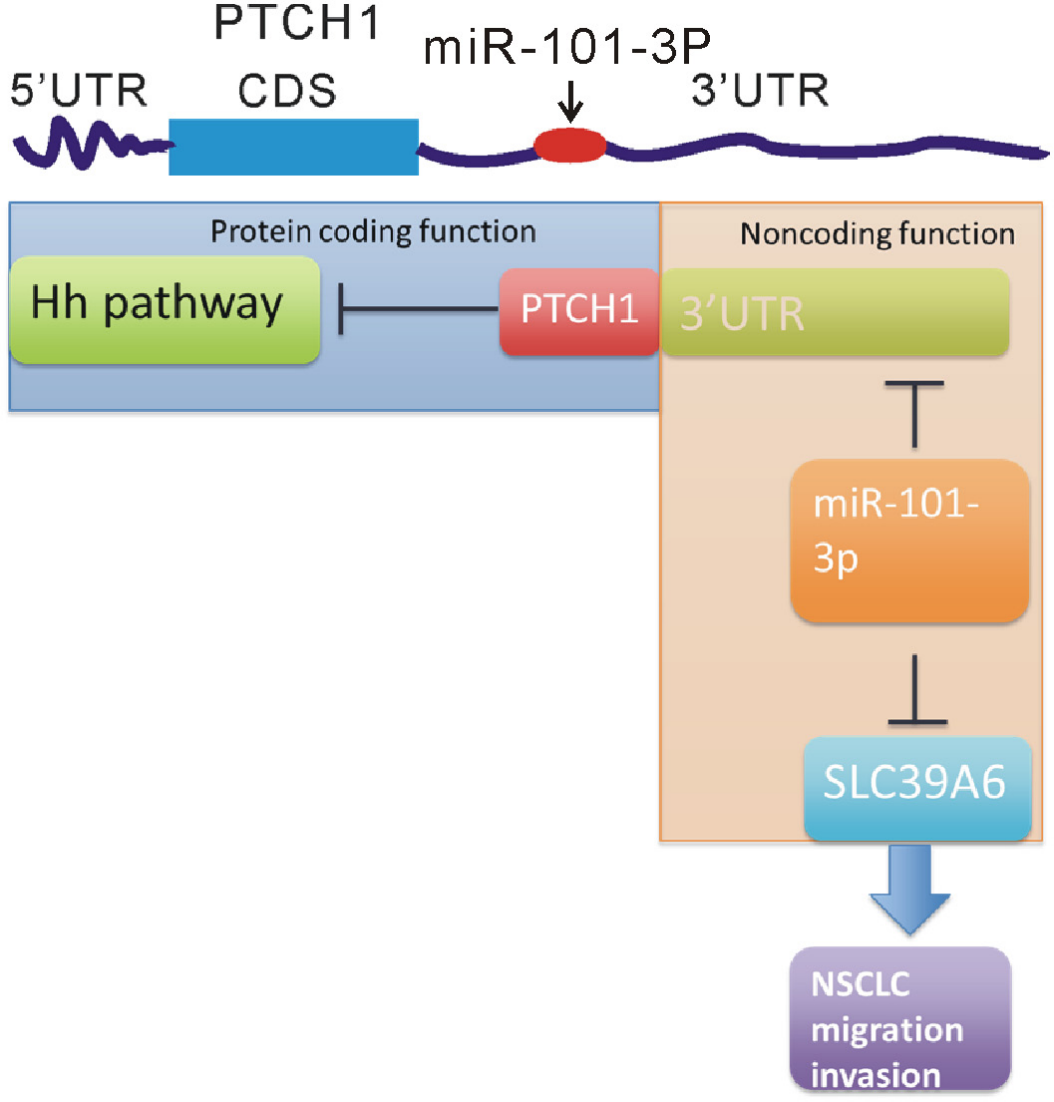
The different roles of the protein and 3’UTR of PTCH1 in regulating NSCLC progression A schematic diagram shows PTCH1 protein inhibited NSCLC cell proliferation via inhibiting Hedgehog (Hh) pathway and PTCH1 3’UTR promoted NSCLC cell migration and invasion by activating miR-101-3p/SLC39A6 axis.

Collectively, our data showed overexpression of PTCH1-3’UTR promoted cell migration, invasion and adhesion, but did not affect cell proliferation in NSCLC cells. Combined WGCNA analysis with experimental validation, we reported a novel mechanism driving metastasis mediated by PTCH1 whose 3’UTR acted as a sponge to absorb miR-101-3p and promoted SLC39A6 expression (Figure 9). Therefore, our results help us to understand the novel function of PTCH1 in NSCLC tumorigenesis and provide novel insights for the prevention of NSCLC metastasis.

## MATERIALS AND METHODS

### Cell culture

All cell lines were obtained from the American Type Culture Collection (Manassas, VA) which were confirmed by short tandem repeat (STR) analysis. All experiments were carried out with cell lines at passages below 30. The medium, fetal bovine serum (FBS), HEPES, nonessential amino acids, and sodium pyruvate were purchased from Invitrogen (Carlsbad, CA). NCI-H1299 and A549 cells were cultured with RPMI 1640 medium supplemented with 10% FBS, 2.383 mg/ml HEPES, and 0.11 mg/ml sodium pyruvate at 37°C in a humidified atmosphere of 5% CO_2_.

### RNA interference, plasmids and transient transfection

Synthetic miR-101-3p mimic, scrambled control miRNA (miR-NC), SLC39A6 siRNA (siSLC39A6), PTCH1 siRNA (siPTCH1) and its negative control (siNC) were purchased from GenePharma (Shanghai, China), and used at the concentration of 50 nM. All sequences of synthetic oligonucleotides are listed in Supplementary Table 1.

The wild-type 3’UTR of PTCH1 was amplified by PCR from H1299 cell line and then inserted into the modified vector. The primers: 5’–CCCATATGTGCCAGGACAGCAGTTCATT-3’ and 5’-CGGAATTCGAGCCTACTACAGGTTACAGACAG-3’ were used. Full-length cDNA of PTCH1-3’UTR was cloned into expression plasmid pcDNA3.1(+) (Invitrogen). Transfection was carried out with Lipofectamine 2000 Transfection Reagent (Life, USA) according to the manufacturer's procedure.

### Construction of gene co-expression network

To explore the interactions between the genes, a system biology approach, WGCNA, which converts co-expression measure into connections weight or topology overlap measure, was applied for gene co-expression network construction [18]. Co-expression methodology was typically used for exploring correlation between gene expression levels. Genes involved in the same pathway or same functional compound tend to demonstrate a similar expression pattern [28]. Therefore, the construction of a gene co-expression network facilitates the identification of genes with similar biological functions [29]. In our work, all of genes of from TCGA data were inputted to construct weighted co-expression modules using the WGCNA package in R language. The threshold of co-expression module was set as P<0.05.

### Microarray and expression data sets

Total RNA from A549 cells, treated with PCDNA3.1(+) or PCDNA3.1(+)-PTCH1-3’UTR for 48 h, were hybridized to Agilent Human lncRNA (4^*^180K, Design ID: 042818). Total RNA of samples was isolated by using TRIzol (Invitrogen) and the RNeasy mini kit (QIAGEN). Total RNA was quantified by the NanoDrop ND-2000 (Thermo Scientific) and the RNA integrity was assessed using Agilent Bioanalyzer 2100 (Agilent Technologies). To begin with, the raw data was normalized with the quantile algorithm. The probes that at least 1 conditions out of 2 conditions had flags in “P” were chosen for further data analysis. Differentially expressed lncRNAs were then identified through fold change. The threshold set for up- and down-regulated genes was a fold change>= 1.5. All results are listed in Supplementary Table 2.

### RNA isolation and real-time qPCR

Total RNA was extracted with Trizol (Invitrogen, CA, USA), and reverse transcription was performed according to the manual of NovoScript^®^ 1^st^ Strand cDNA Synthesis SuperMix (Novoprotein Scientific Inc. China). Real-time quantitative PCR was carried out with AceQ qPCR SYBR Green Master Mix (Vazyme Biotech co., ltd) on LightCycler 480II (Roche, Basel, Switzerland) instrument. Specific primers for mature miR-101-3p were from GenePharma (Shanghai, China). Primers used for qRT-PCR were listed in Supplementary Table 1. The Ct values were normalized using β-actin or RNU6 as internal control to estimate the different expression of genes. Relative mRNA expression was calculated using the 2^-ΔΔCt^ method. Each sample was run in triplicate to ensure quantitative accuracy.

### Western blotting analysis

Cells were lysed in RIPA buffer (Boston Bioproducts) supplemented with protease inhibitors (Complete, EDTA-free; Roche Diagnostics) and PMSF (Calbiochem). Lysates were separated on a 12% acrylamide gel and subjected to western blot analysis. Immunoblots were incubated over night at 4°C with the following primary antibodies: anti-CDH2 (Proteintech, UK), anti-VIM (Proteintech, UK), anti-PTCH1 (Proteintech, UK), anti-SLC39A6 (Proteintech, UK) anti-GAPDH (Proteintech, UK) and anti-Actin antibodies (goat polyclonal; Santa Cruz Biotechnology; 1:2, 000). Goat anti-mouse IgG-HRP and goat anti-rabbit IgG-HRP (Sigma–Aldrich, USA) secondary antibodies were used to visualize bands using Amersham ECL Prime (GE Healthcare, UK).

### Reporter constructs and luciferase assay

600-bp and 671-bp nucleotide sequences corresponding to portion of the 3’-UTR of PTCH1 and SLC39A6, respectively, including the conserved predicted binding site (seed sequence) for miR-101-3p, were inserted into psi-CHECK2 Dual-Luciferase miRNA Target Expression Vector (Promega, USA) within the XhoI/NotI sites. Mutagenesis was performed using Mut Express^®^ II Fast Mutagenesis Kit V2 (Vazyme, USA). All insertions were verified by sequencing. The relative luciferase activity was measured by Dual-Luciferase Reporter Assay System (Promega, USA) 48 h after transfection in H1299 cells (Primer sequences were shown in Supplementary Table 1).

### Cell proliferation assay

Cell proliferation was assessed by Cell Counting Kit-8 (CCK-8, Dojindo Laboratories, Japan) in octuplicate according to the manufacturer's instructions. Absorbance was measured at 450 nm with Microplate Reader ELx808 (Bio-Tek, USA). The absorbance at 615 nm was used as a reference.

### Cell cycle assay

Cells were harvested 48 h after transfection. For cycle assay, cells were incubated with 0.03% triton X-100 and propidium iodide (PI) (50 ng/mL) for 15 min; the percentages of cells in different phases of cell cycle were measured with a FACScalibur flow cytometer (BD, USA) and analyzed with ModFit software (Verity Software House, USA).

### Cell migration and invasion assay

The cell invasion assay was performed in transwell plates (8-μm pore size, 6.5-mm diameter; Corning, USA) precoated with Matrigel Basement Membrane Matrix (coating concentration:1 mg/ml; BD Biosciences, Franklin Lakes, NJ) according to the manufacturer's protocol. Briefly, cells (5 × 10^4^ / well) after transfection were seeded with media containing 1% FBS into the upper chamber of transwell filter on a 24-well plate. Medium containing 10% FBS was used as attractant and added to the lower well of the plate. After 72 hours of incubation, cells on the upper side of the filters were removed and cells migrated to the lower side were fixed with methanol, stained with Giemsa stain, and counted under a microscope. Cells in five randomly chosen fields per transwell filter were counted to quantify the average number of invasion. Migration assays were performed with the same procedure, except that the transwell chamber inserts were not coated with Matrigel, and medium containing 10% FBS was used for the cell suspensions. Each experiment was repeated three times.

### Adhesion assay

The day before seeding, a 96-well plate was coated with 25 μl of matrigel (BD, USA) mixed with 25 μl serum-free 1640 medium (Gibco, USA). About 5,000 cells suspended in 100μl 1640 medium were then seeded into each well of the pre-coated 96-well plate. An hour later, the medium was removed, the cells adhered to matrigel were washed twice with PBS, fixed with methanol for10 min, stained with DAPI, and then photographed with a fluorescence microscope (Olympus, Japan). The number of adherent cells was counted. Each experiment was performed at least in triplicates.

### Statistical analysis

The numerical data were presented as mean ± standard deviation (SD) of at least three determinations. Statistical comparisons between groups of normalized data were performed using T-test or Mann–Whitney U-test according to the test condition. A *p*< 0.05 was considered statistical significance with a 95% confidence level.

## SUPPLEMENTARY FIGURES AND TABLES







## Abbreviations

3’-UTR: 3’-untranslated regions
FBS: fetal bovine serum
miRNA: microRNA
Mut: mutant
NC: negative control
NSCLC: non-small cell lung cancer
PTCH1: patched homolog 1
qRT-PCR: quantitative real time polymerase chain reaction
SLC39A6: solute carrier family 39, member 6
WGCNA: weighted gene correlation network analysis.
