# Supplementary material for: Co-expression analysis revealed PTCH1-3'UTR promoted cell migration and invasion by activating miR-101-3p/SLC39A6 axis in non-small cell lung cancer: implicating the novel function of PTCH1
Source: Oncotarget. 2017 Dec 13;9(4):4798–813. doi: 10.18632/oncotarget.23219 (PMC5797013; doi:10.18632/oncotarget.23219)
Supplement: Supplementary file 1 [file oncotarget-09-4798-s001.pdf]

Co-expression analysis revealed PTCH1-3’UTR promoted cell migration and invasion by activating miR-101-3p/SLC39A6 axis in non-small cell lung cancer: implicating the novel function of PTCH1

SUPPLEMENTARY MATERIALS

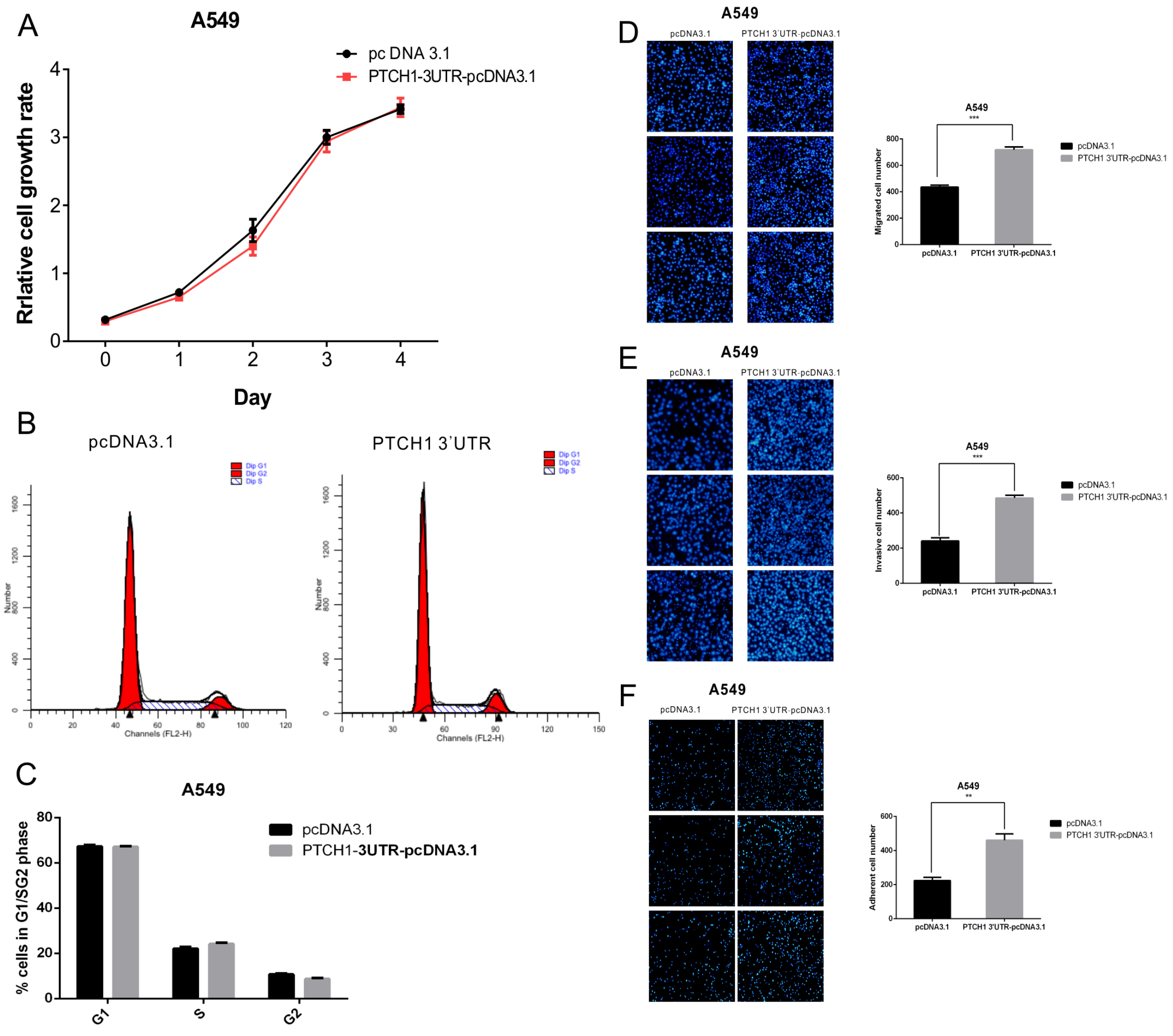

Supplementary Figure 1: Overexpression of PTCH1 3’UTR promotes cell migration, invasion and adhesion in A549. **(A)** Cell proliferation analysis was performed with CCK-8 assay in A549 cells. Cells transfected with PTCH1 3’UTR or pcDNA 3.1 vector were seeded into 96-well plate at 5000 cells/well and examined at time points of 0h, 24h, 48h, 72 h and 96h. Overexpression of PTCH1 3’UTR had no significant difference with that transfected with pcDNA3.1. **(B-C)** Cell cycle assay was performed in A549 cells. Cells were transfected with PTCH1 3’UTR or pcDNA 3.1 vector for 48 h, stained with PI and evaluated with a FACScalibur flow cytometer. Overexpression of PTCH1 3’UTR had almost the same population of cells in the G1 (resting), S (synthesis) and G2 (mitotic) phases with negative control. **(D-F)** Migrations, invasion and adhesion assay were performed in A549 cells. Cells were transfected with PTCH1 3’UTR or control and seeded into the upper chamber of a transwell to count by transwell assay. Overexpression of PTCH1 3’UTR promotes cell migration, invasion and adhesion compared with negative control. Data are presented as the mean ± SD (n = 3). Significance was defined as p<0.05 (*, $p < 0.05$; **, $p < 0.01$; ***, $p < 0.001$).

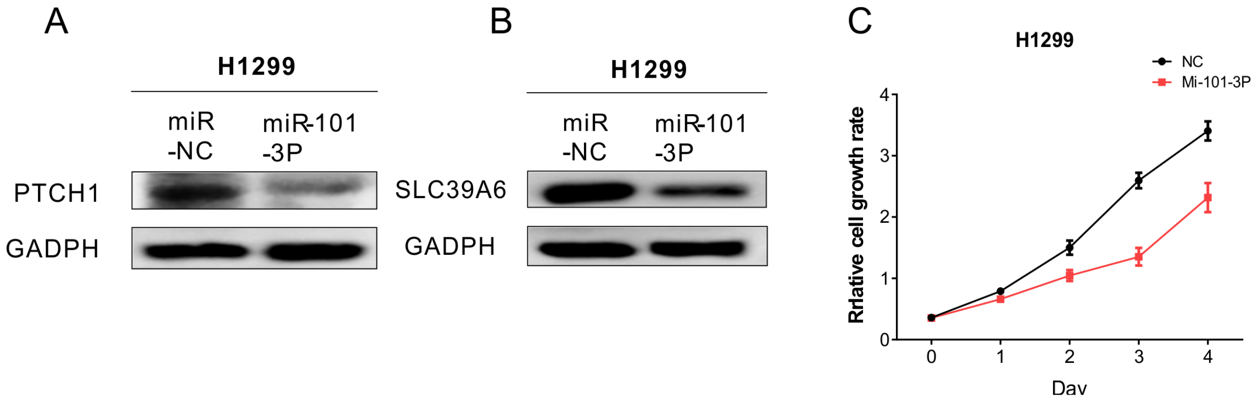

Supplementary Figure 2: Overexpression of miR-101-3p inhibited PTCH1 and SLC39A6 expression and repressed H1299 cell proliferation. **(A-B)** Overexpression of miR-101-3p inhibited PTCH1 and SLC39A6 expression in H1299 cells. **(C)** Overexpression of miR-101-3p inhibited H1299 cell proliferation. Data are presented as the mean ± SD (n = 3). Significance was defined as p<0.05 (*, $p < 0.05$; **, $p < 0.01$; ***, $p < 0.001$).

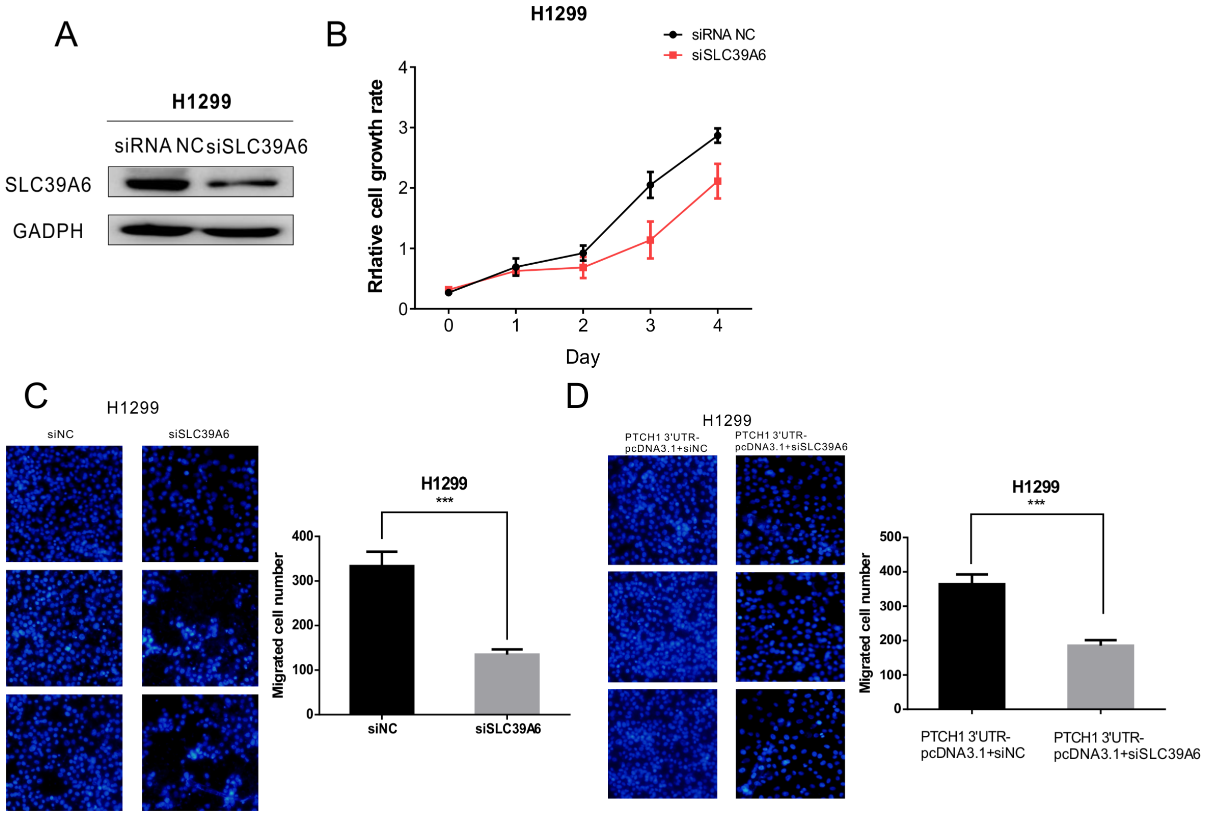

Supplementary Figure 3: PTCH1 3'UTR mediated the effect of SLC39A6 on H1299 migration. (A) Western blot analysis of SLC39A6 expression level after knockdown of SLC39A6 by silencing RNA in H1299. **(B)** Cell proliferation analysis was performed with CCK-8 assay in H1299 cells. Cells transfected with siSLC39A6 or negative control were seeded into 96-well plate at 5000 cells/well and examined at time points of 0h, 24h, 48h and 72h. Knockdown of SLC39A6 significantly suppressed proliferation compared with that transfected with negative control. **(C)** Migrations of H1299 cells after siSLC39A6 transfection were counted by transwell assay, knockdown of SLC39A6 significantly decreased cell migration compared with negative control. **(D)** Migrations of H1299 cells after PTCH1-3'UTR-siSLC39A6 co-transfection were counted by transwell assay, in the presence of PTCH1-3'UTR, the effect of siSLC39A6 on migration could be partially rescued. Data are presented as the mean ± SD (n = 3). Significance was defined as $p<0.05$ (*, $p < 0.05$; **, $p < 0.01$; ***, $p < 0.001$).

Supplementary Table 1: Primers used for ChIP-PCR, RT-PCR, clone, mutation and transfection

	Forward	Reverse
miR-101-3p RT PRIMER	GGCGCCTACAGTACTGTGAT	GTGCAGGGTCCGAGGT
U6 RT PRIMER	CGCTTCGGCAGCACATATACTAA	TATGGAACGCTTCACGAATTTGC
GAPDH RT PRIMER	CTTAGATTTGGTCGTATTGG	GAAGATGGTGATGGGATT
PTCH1 RT PRIMER	CGAAGGTGGAAGTCATTGAGC	CCAACCAGTTCTCTTCAAGCA
SLC39A6 RT PRIMER	GCTGTGTTCTGTCATGAGTTGC	TGCCATTCCAAGATACGCCA
PTCH1 3UTR RT PRIMER	AATAAATATTCAAACTGGGTCTCATTG	TCTTTGTAATTGAAATGACACAAACTC
ACTIN RT PRIMER	CCTCTCCCAAGTCCACACAG	GGGCACGAAGGCTCATCATT
siNC	UUCUCCGAACGUGUCACGUdTdT	ACGUGACACGUUCGGAGAAdTdT
SLC39A6-si-1	CACGGCAAUAUCAUCUACAdTdT	UGUAGAUGAUAUUGCCGUGdTdT
SLC39A6-si-2	GGCUUAUCAAGUGGUUUAAdTdT	UUAAACCACUUGAUAAGCCdTdT
SLC39A6-si-3	CACAGGAAGUCUACAAUGAdTdT	UCAUUGUAGACUUCCUGUGdTdT
miR-101-3p mimics	UACAGUACUGUGAUAACUGAA	UUCAGUUAUCACAGUACUGUA
PTCH1 siRNA	GAUUGGAGAAGAGGCUAUGUU	CAUAGCCUCUUCUCCAAUCUU
PTCH1 3'UTR clone	GAAGATCCGCAAAGAGGCCAAAGATTGGAAACCC	GCAGGAATTCTFFCTCCAACACTAACTGTCTCTCCC
3' UTR SLC39A6	GCAGTAATTCTAGGCGATCGCTCGAGTAGTTTCAGTAGGTCATAGGGAGA	AAAGATATTTTATTGCGGCCAGCGGCCGCGAGAATGCCACAGACAGG
3' UTR PTCH1	GCAGTAATTCTAGGCGATCGCTCGAGGGGTGATTAAAATCTGAAGCAAAGA	AAAGATATTTTATTGCGGCCAGCGGCCGCACTCTGGGGCAGGAGTGGAG
3' UTR PTCH1-MUT	CATTGTatgacaAACCGATTGTATTATTTTGTTAAATATTTC	TCGGTTtgtcatACAATGAACTGCTGTCCTGGCA
3' UTR SLC39A6-MUT	ATACGacgtacgAGCCATACTAGGCCTGTCTGTGG	TGGCTcgtacgtCGTATATAAAAGACAATTGCTCACAATG

Supplementary Table 2: The differently expressed mRNAs in A549 cells after the overexpression of PTCH1-3’UTR

See Supplementary File 1

Supplementary Table 3: A complete list of the network metrics and the module membership for each gene

See Supplementary File 2
